# RES-Seq—a barcoded library of drug-resistant *Leishmania donovani* allowing rapid assessment of cross-resistance and relative fitness

**DOI:** 10.1128/mbio.01803-23

**Published:** 2023-11-06

**Authors:** Lindsay B. Tulloch, Sandra Carvalho, Marta Lima, Richard J. Wall, Michele Tinti, Erika G. Pinto, Lorna MacLean, Susan Wyllie

**Affiliations:** 1 Wellcome Centre for Anti-Infectives Research, School of Life Sciences, University of Dundee, Dundee, United Kingdom; 2 Drug Discovery Unit, Wellcome Centre for Anti-infectives Research, University of Dundee, Dundee, United Kingdom; Rutgers-New Jersey Medical School, Newark, New Jersey, USA

**Keywords:** drug resistance profiling, mechanism of action, *Leishmania*, visceral leishmaniasis, Illumina sequencing

## Abstract

Visceral leishmaniasis (VL) is a parasitic disease endemic across multiple regions of the world and is fatal if untreated. New therapeutic options with diverse mechanisms of actions (MoAs) are required to consolidate progress toward control of this disease and combat drug resistance. Here, we describe the development of a scalable resistance library screen (RES-Seq) as a tool to facilitate the identification and prioritization of anti-leishmanial compounds acting via novel MoA. We have amassed a large collection of *Leishmania donovani* cell lines resistant to frontline drugs and compounds in the VL pipeline, with resistance-conferring mutations fully characterized. New phenotypic hits screened against this highly curated panel of resistant lines can determine cross-resistance and potentially shared MoA. The ability to efficiently identify compounds acting via previously established MoA is vital to maintain diversity within drug development portfolios. To expedite screening, short identifier DNA barcodes were introduced into resistant clones enabling pooling and simultaneous screening of multiple cell lines. Illumina sequencing of barcodes enables the growth kinetics and relative fitness of multiple cell lines under compound selection to be tracked. Optimal conditions allowing discrimination of resistant and sensitive clones were established (3× and 10× EC_50_ for 3 days) and applied to screening of a complex library with VL preclinical and clinical drug candidates. RES-Seq is set to play an important role in ensuring that anti-leishmanial compounds exploiting diverse mechanisms of action are developed, ultimately providing options for future drug combination strategies.

**IMPORTANCE:**

Visceral leishmaniasis (VL) remains the third largest parasitic killer worldwide, responsible for 20,000–30,000 deaths each year. Control and ultimate elimination of VL will require a range of therapeutic options with diverse mechanisms of action to combat drug resistance. One approach to ensure that compounds in development exploit diverse mechanisms of action is to screen them against highly curated cell lines resistant to drugs already in the VL pipeline. The identification of cross-resistant cell lines indicates that test compounds are likely acting via previously established mechanisms. Current cross-resistance screens are limited by the requirement to profile individual resistant cell lines one at a time. Here, we introduce unique DNA barcodes into multiple resistant cell lines to facilitate parallel profiling. Utilizing the power of Illumina sequencing, growth kinetics and relative fitness under compound selection can be monitored revolutionizing our ability to identify and prioritize compounds acting via novel mechanisms.

## INTRODUCTION

Visceral leishmaniasis (VL), also known as kala-azar, is a disease caused by infection with the protozoan parasites *Leishmania donovani* or *L. infantum*. Infection is mediated through the bite of the female phlebotomine sandfly, and once transmitted to the human host, *Leishmania* spp. parasitize host macrophages. VL disproportionately impacts impoverished communities in Asia, East Africa, and South America, where between 50,000–90,000 new cases and 20,000–30,000 deaths are reported every year (1). In 95% of cases, clinically symptomatic VL patients will die without therapeutic intervention; however, current treatment options for VL are far from ideal. Miltefosine and liposomal amphotericin B (AmBisome) are considered as frontline therapies. While both drugs are superior to previous treatments such as pentavalent antimonials (2), they suffer from serious limitations. The principal drawbacks of miltefosine are teratogenicity, prolonged treatment regimen, and high resistance potential (3). Problems associated with amphotericin B include high treatment costs, an intravenous route of administration, requirement for a cold chain, and unresponsiveness in some Sudanese VL patients (4). Thus, new, effective, and fit-for-purpose drugs are still required.

A severe lack of robustly validated drug targets in *Leishmania* spp. has limited target-focused drug discovery for VL, leaving drug discovery programs reliant upon whole-cell (phenotypic) screening to identify chemical start points (5). This strategy can be effective but is associated with disproportionately high rates of attrition. Perhaps, the principal drawback of phenotypic drug discovery is the difficulty in evolving chemical series to overcome obstacles such as poor pharmacokinetic properties or toxicity without knowledge of the molecular target. Indeed, the majority of successful drug discovery programs find ways to combine knowledge of the target alongside cell-based screening to identify drug candidates. The development of several recent VL drug candidates has been greatly assisted by comprehensive mechanism of action (MoA) studies carried out in concert with medicinal chemistry (6–9). In these particular cases, knowledge of the target during development allowed toxic liabilities associated with the target itself to be directly assessed, enabling more selective compounds to be prioritized (10). Understanding MoA at a relatively early stage of the drug discovery process can also prevent development pipelines from becoming overpopulated with compounds acting against the same molecular targets. This has been a particular issue for kinetoplastid drug discovery with multiple phenotypic hits found to interact with the same small number of molecular targets, specifically CYP51 (11), cytochrome *bc1* (12, 13), and the proteasome (6, 8, 14, 15). The most robust pipelines comprised chemical series that target a diverse range of molecular targets; this spreads any risk that might be associated with specific targets and also provides more options for future drug combination therapies.

*In vitro* evolution of resistant parasites followed by whole-genome sequencing (WGS) is one of the most commonly used approaches to identify the molecular targets of phenotypically active compounds. Our standard approach involves exposing clonal, drug-sensitive parasites to stepwise increasing compound concentrations until they readily grow at concentrations that were previously cidal (7, 12, 16). “Resistant” parasites are then cloned by limiting dilution, genomes sequenced and compared to the genomes of drug-sensitive parasites in order to identify genomic changes (copy number variations and/or single-nucleotide polymorphisms) that may reveal the compound’s molecular target. Putative targets identified through this approach are then validated using secondary approaches such as CRISPR-Cas9 gene editing (17) or target overexpression (16). Once resistant cell lines have been comprehensively characterized, they can become an invaluable resource in future MoA studies. Newly identified, phenotypically active compounds can be screened against these panels of resistant cell lines to allow identification of those acting via previously identified MoA. This can be a rapid and highly efficient way of profiling new hits to preserve diversity of mechanism and molecular targets within drug discovery portfolios.

Over the last 10 years, we have assembled a large collection of highly curated *L. donovani* cell lines resistant to a broad range of compounds with diverse mechanisms of action. Here, we describe the process of introducing unique DNA barcodes into each resistant cell line to facilitate parallel profiling to assess drug resistance, sensitivity, and relative fitness. Barcodes were introduced into parasites enabling multiple cell lines to be pooled and grown together in a single library under compound selection. Following selection, the composition of the library is determined by PCR amplification of barcodes followed by next-generation sequencing (NGS). This scalable resistance library screen (RES-Seq) has revolutionized our ability to identify phenotypically active compounds acting via previously established MoA.

## RESULTS AND DISCUSSION

### Introducing unique identifier barcodes into *L. donovani* drug-resistant clones

*In vitro* evolution of resistant parasites followed by WGS to identify resistance-conferring genomic changes is a standard approach employed to identify the molecular targets of phenotypically active compounds. Over the last few years, we have amassed a large collection of drug-resistant *L. donovani* clones that have been extensively profiled to identify the genomic changes driving resistance. This highly curated panel of resistant cell lines has proved an invaluable resource for our ongoing MoA studies since new anti-leishmanials can be screened against these parasites to determine cross-resistance and potentially shared MoA. The ability to efficiently identify compounds acting via previously established mechanisms of action is vital since it supports drug discovery programs to maintain diversity in their drug development portfolios, thus enabling the limited resources available for VL drug discovery to achieve maximum impact.

To expedite screening of resistant cell lines, we introduced short identifier DNA sequences, or “barcodes,” into each resistant cell line, enabling pooling of these clones and simultaneous screening for cross-resistance. In designing the format of our identifier barcodes, our principal aims were to ensure the accuracy of subsequent cell line identification and scalability of the library in the future. With this in mind, we selected to use 7 bp barcodes, theoretically permitting the generation of up to 16,384 (4^7^) unique barcodes. Barcodes bearing features that could prove problematic for subsequent cloning or sequencing were removed, yielding a final total of 6,350 useable barcodes. Since “reading” barcodes requires a PCR amplification step, a second 7-bp barcode (variable 2) was introduced downstream of variable 1 with the rationale that the likelihood of PCR-mediated mutations occurring in both barcodes and leading to cell line mis-identification would be extremely low. Complementary forward and reverse barcodes were hybridized and then cloned into the multiple cloning site of the *Leishmania*-specific plasmid pIR-SAT (18) (Fig. S1).

### Library generation and assessment of growth kinetics

To establish proof of concept for our barcoding strategy, plasmids containing unique barcodes were transfected as episomes into *L. donovani* wild-type (WT) promastigotes and 12 clonal cell lines resistant to several experimental or clinically used anti-leishmanials (Table 1; Table S1). These cell lines were resistant to compounds known to inhibit a range of molecular targets including the proteasome, oxidosqualene cyclase (OSC), the cyclin-dependent kinase CRK12, and cytochrome *b* (summarized in Table 1; Tables S2 and S3). In each case, the mutations driving resistance in these selected cell lines have been fully characterized (summarized in Table 1). Promastigotes successfully transfected with barcode constructs were pooled into a single culture, with each resistant cell line equally represented. The combined culture was diluted to a cell density of 1 × 10^5^ cells/mL.

**TABLE 1:** Properties of *L. donovani* cell lines used in this study

Cell line	Compound used in resistance evolution	Target implicated in resistance (gene ID)	Resistance conferring mutations/gene amplifications/deletions	POC screen	Expanded library screen	References
DDD01012248-Res 3	DDD01012248	β5 Proteasome subunit (LdBPK_361730.1)	Target mutation (G197S)	*	*	(6)
DDD00107332-Res 1	DDD00107332	CRK12 (LdBPK_090270.1)	Target mutation (G572D)/amplification	*	*	(7)
CRK12^G572D^	–	CRK12 (LdBPK_090270.1)	Target mutation (G572D)		*	(19)
Amph B-Res 1	Amphotericin B	SMT (LdBPK_362510.1)	Resistance-associated deletion	*	*	–
Amph B-Res 3	Amphotericin B	P450R (LdBPK_281350.1)	Resistance-associated INDEL	*	*	–
GSK2920487A-Res 2	GSK2920487A	OSC (LdBPK_060670.1)	Target amplification	*	*	(16)
DNDI-6690-Res 2	DNDI-6690	DNM1 (LdBPK_292310.1)	Resistance-associated mutation (G606V)	*	*	(20)
DNM1^G606V^	–	DNM1 (LdBPK_292310.1)	Resistance-associated mutation (G606V)		*	–
CPSF3^N219H^	–	CPSF3^*a*^ (LdBPK_343210.1)	Target mutation (N219H/E229V)		*	(17)
DDD01716002-Res 1	DDD01716002	Cytochrome *b* (kinetoplast)	Target mutation (S207P)	*	*	(12)
DDD01716002-Res 2	DDD01716002	Cytochrome *b* (kinetoplast)	Target mutation (G31A)	*	*	(12)
DDD01716002-Res 3	DDD01716002	Cytochrome *b* (kinetoplast)	Target mutation (F227I)	*	*	(12)
BPQ-Res 3	Buparvaquone	Cytochrome *b* (kinetoplast)	Target mutation (F147V)	*	*	
DDD01542111-Res 1	DDD01542111	Cytochrome *b* (kinetoplast)	Target mutation (G37A)	*	*	(12)
DDD01542111-Res 3	DDD01542111	Cytochrome *b* (kinetoplast)	Target mutation (C222F)	*	*	(12)
NTR2 DKO	–	NTR2 (LdBPK_120730.1)	Pro-drug activator deletion		*	(21)
Fexinidazole-Res C	Fexinidazole	NTR1 (LdBPK_050660.1)	Pro-drug activator functional deletion		*	(22)

^*a*^ CPSF3, cleavage and polyadenylation specific factor 3.

Mindful that resistance-conferring mutations can impact the general fitness of parasites, the relative growth kinetics of each barcoded cell line within the proof-of-concept library was monitored in the absence of drug selection. Growth of the composite library was monitored over 9 days, with samples collected for genomic DNA isolation on days 0, 3, 6, and 9. The culture was continued for a further 19 days without continued monitoring of growth, and a snapshot sample was harvested for genomic DNA preparation at that time. Following genomic DNA extraction, barcodes harbored by each cell line in the culture were amplified by PCR.

**Fig 1:**
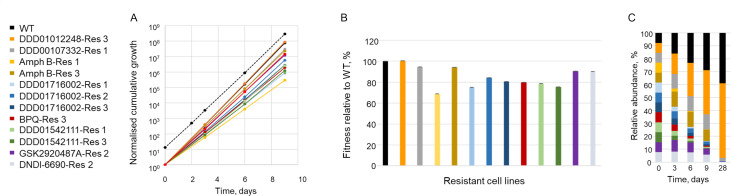
Growth kinetics of individual cell lines within the pooled proof-of-concept library. (**A**) Normalized growth of the composite library based on cell density (black-dotted line). (**B**) Relative fitness of each cell line within the library relative to WT. The “fitness” of each cell line was calculated using the reciprocal doubling time and expressed as a percentage relative to WT (100%). Each data point illustrates the mean ± SD for triplicate measurement. (**C**) Composition of the library over time. Color-coded key to identify each cell line within the library provided.

To maximize the granularity and resolution of the data generated from our proof-of-concept library, we utilized the power of NGS (Illumina) to analyze PCR-generated amplicons. Reads were aligned to barcodes, and the number of barcodes for each cell line was scored. Total reads containing a barcode ranged from 0.13 to 1.6 million reads per selection condition, with an average of 0.44 million reads. Barcode reads were then used to determine the composition of the library at defined intervals during compound selection and their relative abundance quantified. NGS data sets generated from screening of the proof-of-concept library are detailed in Extended data set I. The relative growth rates of individual cell lines within the library were established by measuring the change in relative abundance of barcodes over time. As expected, individual cell lines within the library grew at visibly different rates (Fig. 1A). The doubling time of each cell line was calculated and found to closely mirror those previously determined in individual culture (Table S4), indicating that combining cell lines did not significantly impact their growth kinetics. Next, the fitness of each cell line was calculated using the reciprocal doubling time and expressed as a percentage relative to WT (100%) (Fig. 1B). Of particular note, the relative fitness of DDD01012248-Res 3 (101%), a cell line bearing a mutation in the β5 subunit of the proteasome and resistant to the specific proteasome inhibitor DDD01012248 (6), marginally exceeded that of the WT, parental cell line. In contrast, Amph B-Res 1, resistant to the standard of care anti-leishmanial amphotericin B, demonstrated markedly reduced fitness compared to WT (69%). Variations in doubling times and relative fitness changed the composition of the library over time (Fig. 1C). Faster growing cell lines such as WT and DDD01012248-Res 3 became enriched and, by day 28, dominated the library. While barcodes representing all of the cell lines originally seeded into the library were detectable, even in the day 28 culture, to maintain the complexity of the library going forward, we limit the number of library passages to a maximum of 3, equivalent to 9 days in culture (without drug). Pleasingly, the relative fitness of individual-barcoded cell lines in this library iteration, as well as in expanded libraries described later, was highly reproducible across multiple screens (Table S5).

### Compound selection of the proof-of-concept library

**Fig 2:**
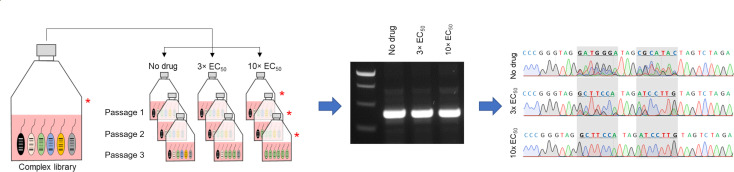
Overview of the proof-of-concept library screening workflow. Uniquely barcoded resistant cell lines were pooled to form a single barcoded library. The complex library was split into three and grown for a maximum of three passages in the absence of compound selection, with compound treatment at 3× or 10× the established EC_50_ value. Samples of each culture were harvested at time 0 and prior to passage 2, 3, and 4 (denoted by asterisk). Genomic DNA was extracted from each culture, barcodes were amplified by PCR, and then “read” by Sanger or Illumina sequencing.

**Fig 3:**
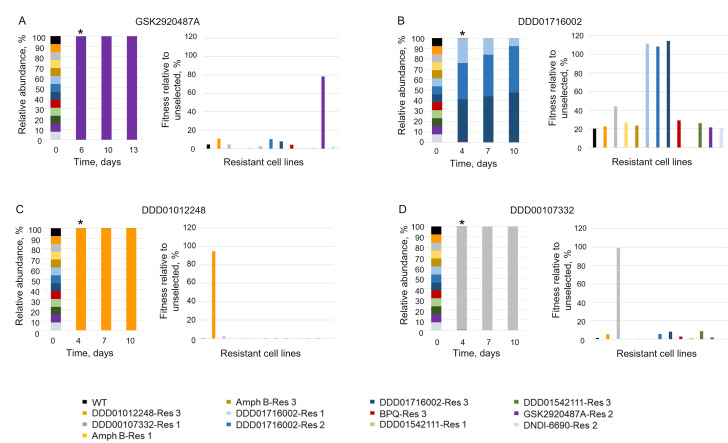
Compound selection of the proof-of-concept barcoded library. Library composition (%) over time and fitness profiling of cell lines (relative to unselected) following selection (one passage) with GSK2920487A (**A**), DDD01716002 (**B**), DDD01012248 (**C**), and DDD00107332 (**D**) at 10× their respective EC_50_ values. Color-coded key to identify each cell line within the library provided. Asterisk added to each bar chart to denote the point where relative cell line fitness was calculated.

To assess the utility of this library in identifying parasites resistant to drug selection, the proof-of-concept library was screened with five compounds. Four of the compounds screened were used in the evolution of resistant cell lines within the library and thus acted as positive control compounds (DDD01012248, DDD00107332, DDD01716002, and GSK2920487A; structures of all compounds used in this study are shown in Fig. S2). The remaining compound (DDD85646) is an established inhibitor of *N*-myristoyltransferase (NMT) in *L. donovani* (23), and since none of the cell lines in the proof-of-concept library were expected to be resistant to NMT inhibitors, we considered DDD85646 our negative control compound. In each case, the library was exposed to the test compounds at concentrations equivalent to 3× and 10× their respective WT EC_50_ values (Table S2). An unselected library was also grown in parallel. The libraries were selected over three passages (9–12 days), with genomic DNA harvested after every passage and at day 0 (Fig. 2). In this instance, the relative fitness of each cell line was calculated using the reciprocal doubling time and expressed as a percentage relative to the unselected line (100%). Selection of the library with GSK2920487A (OSC inhibitor) (16), DDD00107332 (CRK12 inhibitor) (7), and DDD01012248 (proteasome inhibitor) (6) at 10× their established EC_50_ values resulted in the rapid enrichment of cell lines known to be resistant to these compounds, with selection completed by the end of the first passage (Fig. 3). Indeed, under drug selection, these cell lines demonstrated vastly superior fitness compared to others within the library. Illustrating the power and resolution of NGS, the enrichment and enhanced relative fitness of all three DDD01716002-resistant cell lines were clearly evident in libraries exposed to DDD01716002 at both 3× and 10× EC_50_. In general, libraries exposed to test compounds at 3× EC_50_ comprised mixed parasite populations; nevertheless, the majority of barcode reads were still associated with the expected resistant cell lines (Fig. S3). In library cultures exposed to our negative control compound (DDD85646) at 10× EC_50_, no viable parasites were visible after 4 days. NGS data indicated that the cell line DNDI-6690-Res 2 was significantly enriched following treatment with DDD85646 at 3× EC_50_; however, the relative fitness of these parasites during selection was confirmed as no better than others within the library (Fig. S4). These data confirm the absence of cell lines truly resistant to DDD85646 in our proof-of-concept library and emphasize the value of relative fitness assessment in interpreting NGS data.

Collectively, these data confirm the utility of our library to successfully identify resistant and cross-resistant cell lines. In addition, these preliminary data indicate that in libraries exposed to relatively modest drug pressure, the fittest and fastest-growing cell lines will be enriched alongside high- and low-level-resistant parasites. Library selection at 10× EC_50_ enables high-level-resistant cell lines to be efficiently identified but may lead to subtle but informative responses to compound treatment being missed. Thus, we opted to continue screening at both 3× and 10× EC_50_ to gain maximum insight.

### Library expansion and screening of VL preclinical and clinical candidates

Following *in vitro* resistance selection with compounds of interest, mutations that we suspect are involved in conferring resistance or target related are validated using a range of genetic approaches including gene editing via CRISPR-cas9 or other alternatives, gene knockout, or target overexpression. We rationalized that barcoding these genetically engineered cell lines and adding them to our library may provide increased confidence in identifying cross-resistance and further insight into target-related factors driving cross-resistance. It should be noted that not all mutations within cell lines generated *in vitro* are specifically related to the compound target. In the worst-case scenario, these off-target, non-specific mutations can erroneously link the mechanisms of action of two compounds. Simultaneous screening of genetically modified parasites engineered in a WT background can prevent this from happening. With this in mind, a number of genetically modified cell lines were added to the second version of our library (Table 1; Table S1).

**Fig 4:**
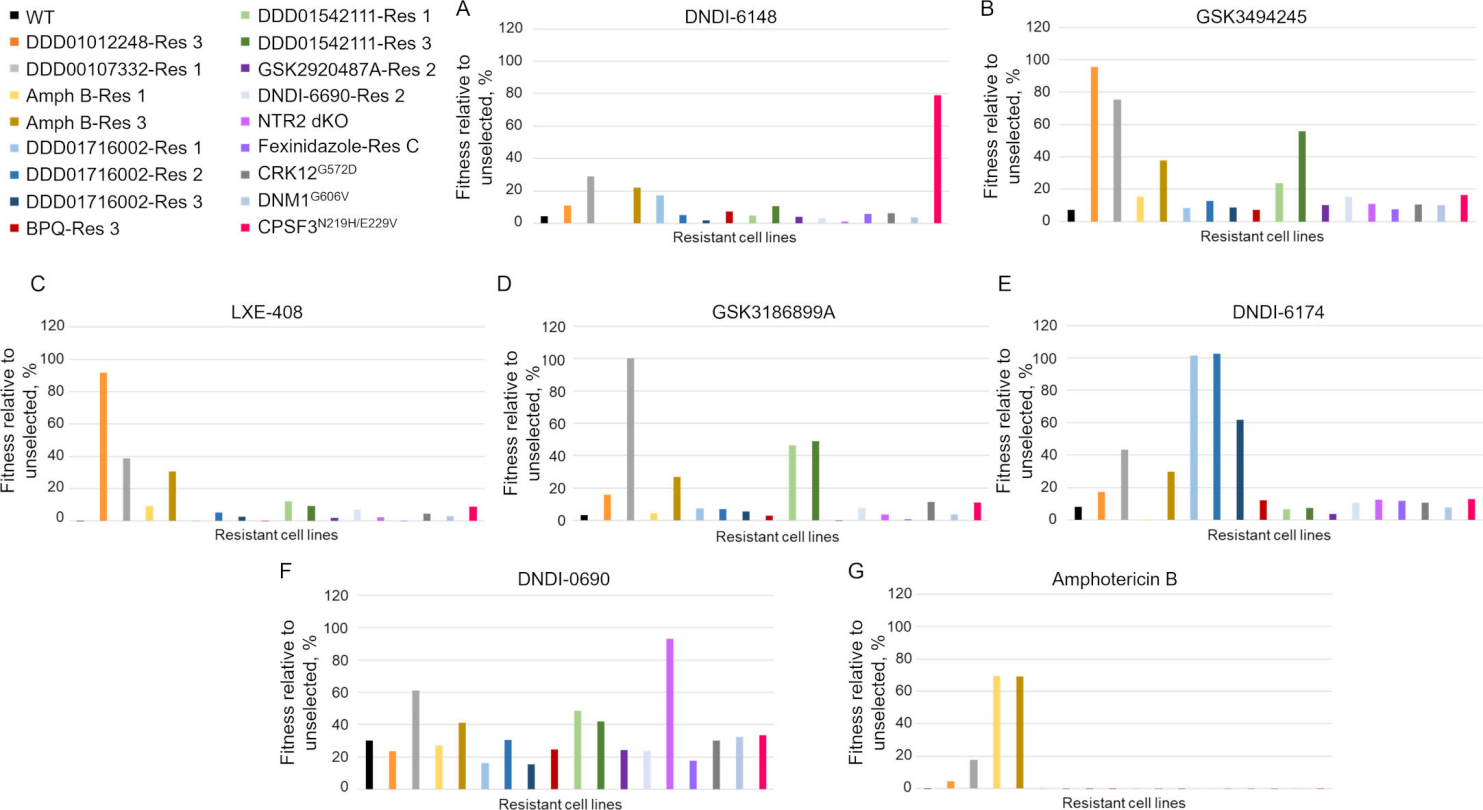
Selection of the expanded barcoded library with VL clinical and preclinical drug candidates. Fitness profiling of cell lines within the library relative to unselected cell lines following selection (one passage) with DNDI-6148 (**A**), GSK3494245 (**B**), LXE-408 (**C**), GSK3186899A (**D**), DNDI-6174 (**E**), DNDI-0690 (**F**), and amphotericin B (**G**) at 10× their respective EC_50_ values. Color-coded key to identify each cell line within the library provided. Asterisk added to each bar chart to denote the point where relative cell line fitness was calculated.

Next, we screened our expanded library with the clinical candidates currently in development for the treatment of VL with a view to identifying the most, or least, appropriate compounds for future combination therapies. Our previous proof-of-concept screen demonstrated that exposing the library to compound treatment for a single passage (typically 3 days) generated sufficient data to discriminate between sensitive and resistant cell lines. Thus, seven clinical and preclinical drug candidates, as well as three current frontline anti-leishmanials, were screened against the expanded library at 3× and 10× their respective EC_50_ values for a single passage. Genomic DNA for subsequent NGS analysis was prepared from each culture, as well as the starter culture at day 0. NGS data sets generated from compound screening of the expanded library are detailed in Extended data set II. As in our proof-of-concept screen, fitness profiling of cell lines exposed to compounds at 10× EC_50_ enabled resistant clones to be definitively and clearly identified (Fig. 4). Selection of the library with DNDI-6148, known to target the cleavage and polyadenylation specific factor 3 in *L. donovani* (17), successfully enriched a CRISPR-edited cell line bearing specific mutations (N219H and E229V) in this enzyme involved in RNA processing and maturation. Similarly, screening of two clinical candidates (GSK3494245 and LXE-408) that target the interface of the β4/β5 subunits of the proteasome enriched DDD01012248-Res 3, a cell line with a mutation in this specific region. GSK3186899A, a clinical candidate and close analog of DDD00107332, enriched DDD00107332-Res 1, a cell line known to overexpress a mutated version of CRK12 (^G572D^) as well as the partner cyclin of this kinase, cyclin 9. Interestingly, an oligo-edited cell line where the G572D mutation was engineered in CRK12, without accompanying overexpression, was only enriched in the GSK3186899A-selected library at 3× but not 10× EC_50_ (Fig. S5), indicating that overexpression of this kinase and its accompanying cyclin is the primary driver for resistance. As expected, DNDI-6174, which targets the *Q*_*i*_ site of cytochrome *b* (manuscript in review), enriched all three resistant cell lines evolved through selection with the established *Q*_*i*_ site inhibitor DDD01716002 (12). Indeed, cross-resistance between DNDI-6174 and DDD01716002 has been previously reported (manuscript in review). In contrast, DNDI-6174 did not select any of the cell lines resistant to another cytochrome *b* inhibitor, DDD01542111, nor the cytochrome *b Q*_*o*_-site inhibitor buparvaquone. We have previously reported that both *Q*_*i*_-site mutations carried by DDD01542111-resistant parasites render them hyper-sensitive to DDD01716002 (12). Thus, the resistance profile of DNDI-6174 closely mimics that of DDD01716002, indicating that these compounds may exploit similar binding pockets. More broadly, these data illustrate the great value of building a complex library consisting of cell lines representing a full complement of target-specific mutations.

Previous studies from our lab confirmed that the bicyclic nitroaromatic compound DNDI-0690 is a prodrug that requires activation by the nitroreductase NTR2 for activity (21). In keeping with these findings, our NTR2-null cell line retained almost 100% of its relative fitness in the DNDI-0690-selected library (10× EC_50_). However, a cell line generated through exposure to the monocyclic nitro drug fexinidazole, exclusively activated by the nitroreductase NTR1, did not retain fitness during selection with DNDI-0690. These data suggest that there may be little or no functional redundancy between these two nitroreductases and that their substrate specificities are likely to be markedly different.

The expanded library was then screened with drugs currently used in the clinic for the treatment of VL. Reassuringly, parasites within the library were unable to survive selection with the alkyl phosphocholine drug miltefosine at either 3× or 10× EC_50_. In libraries selected with the current standard of care VL therapy amphotericin B, only resistant cell lines previously evolved through *in vitro* exposure to amphotericin B retained their fitness (Fig. 4; Fig. S5). Despite associated toxicity and the emergence of wide-spread drug resistance, pentavalent antimonials remain in clinical use for the treatment of both VL and cutaneous leishmaniasis. Pentavalent antimonials act as prodrugs that require reduction to trivalent forms for anti-leishmanial activity. The mechanism of bio-activation is not fully understood but occurs exclusively in the mammalian, intracellular stages of parasites infection. Since we screen our library at the insect, promastigote stage, we opted to assess potential antimonial cross-resistance through selection with a trivalent form (potassium antimonyl tartrate). While cell lines within the library were able to tolerate potassium antimonyl tartrate at 3× EC_50_, none of the lines retained significant fitness (Fig. S5), and no parasites survived selection at 10× EC_50_. Collectively, these data indicate that current preclinical/clinical VL candidates and drugs in clinical use do not share mechanisms of resistance, at least those represented in this library. This is vitally important since the target product profile for VL states that all future therapies must be capable of treating all strains of *Leishmania* resistant to existing drugs (24).

### Reciprocal cross-resistance between inhibitors of CRK12 and the cytochrome *b* inhibitor DDD01542111

**Fig 5:**
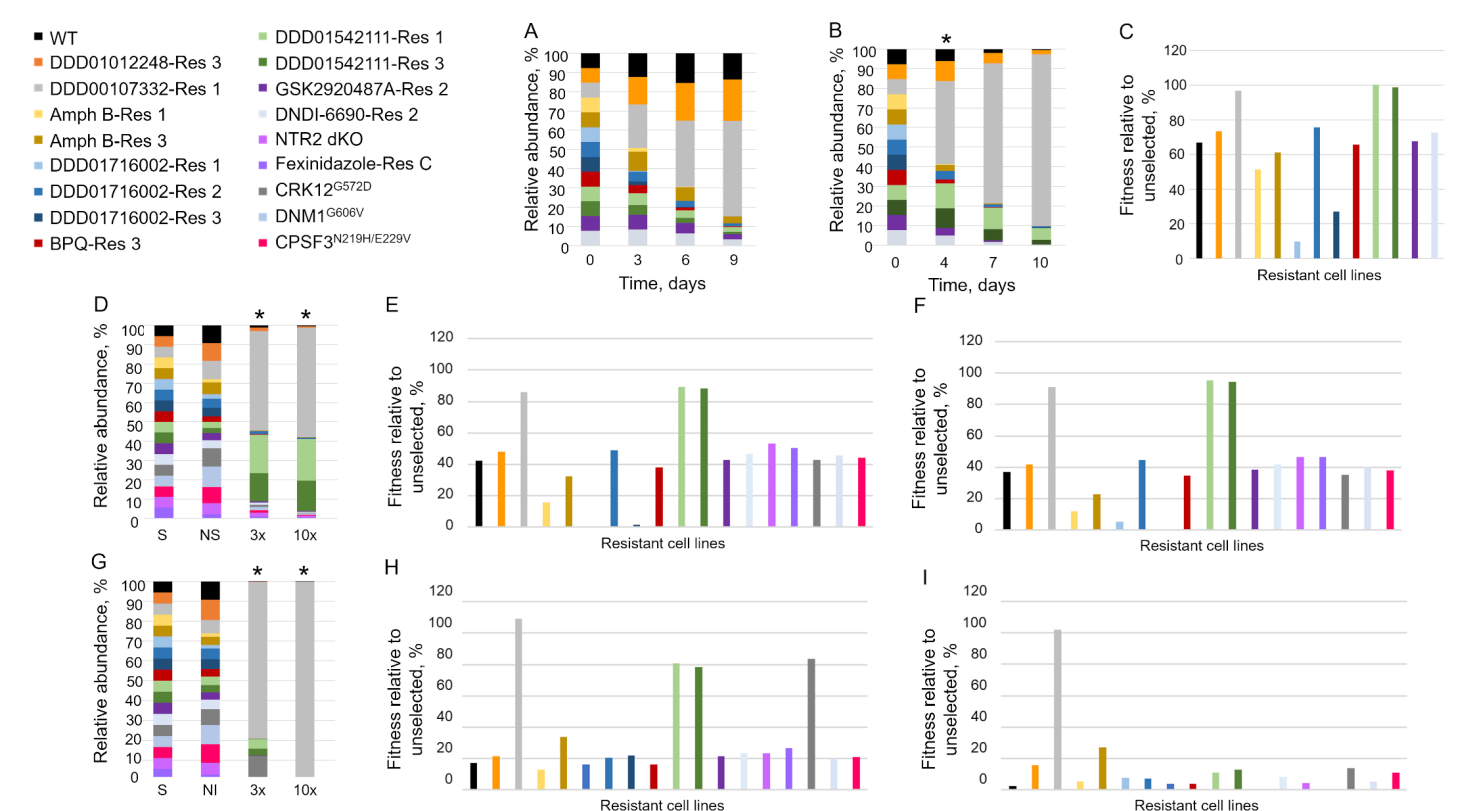
Cross-resistance between cell lines resistant to CRK12 and cytochrome b inhibitors. Proof-of-concept library composition (%) over time following selection with at 3× (**A**) and 10× (**B**) the established EC_50_ value of DDD01542111. (**C**) Fitness profiling of cell lines within the proof-of-concept library (relative to unselected) following selection (one passage) with DDD01542111 at 10× EC_50_. Expanded library composition (%) over time following selection with DDD01542111 (**D**) and DDD00107332 (**G**) at 3× and 10× their respective EC_50_ values. Fitness profiling of cell lines within the expanded library relative to unselected cell lines following selection (one passage) with DDD01542111 and DDD00107332 at 3× (**E and H**) and 10× (**F and I**) their respective EC_50_ values. Color-coded key to identify each cell line within the library provided. Asterisk added to each bar chart to denote the point where relative cell line fitness was calculated.

One unexpected finding from our initial proof-of-concept library screen was the apparent fitness of cell lines resistant to the CRK12 inhibitor DDD00107332 during selection with the cytochrome *b* inhibitor DDD01542111 (Fig. 5A through C). Indeed, in the library selected at 10× EC_50_, the relative fitness of the cell line DDD00107332-Res 1 (97%) was equivalent to that of DDD01542111-Res 1 (100%) and 3 (99%). Both DDD01542111-resistant lines also demonstrated enhanced fitness (76% and 66%, respectively) during selection with DDD00107332 (3× EC_50_). Importantly, this reciprocal cross-resistance relationship was confirmed by follow-up EC_50_ determinations with cell lines in individual culture (Table S6).

To further investigate this unexpected association, both DDD00107332 and DDD01542111 were screened against our expanded library (Fig. 5). In keeping with our earlier observation, in libraries selected with DDD01542111 at 3× and 10× EC_50_, both DDD00107332- and DDD01542111-resistant promastigotes demonstrated enhanced fitness profiles. In contrast, the cell line bearing the oligo-edited G572D mutation in CRK12 was not enriched during either selection condition. As expected, in the library selected with DDD00107332 (3× EC_50_), the oligo-edited and DDD00107332-resitant cell lines demonstrated enhanced fitness. In addition, both DDD01542111-resistant cell lines retained fitness, although this cross-resistance phenotype was not evident when the library was selected at 10× EC_50_.

Next, we took a closer look at the libraries selected with the CRK12-targeting clinical candidate GSK3186899A (Fig. 4D; Fig. S5D) and the cytochrome *b*-targeting preclinical candidate DNDI-6174 (Fig. 4E; Fig. S5E). DD01542111-resistant cell lines demonstrated enhanced fitness in libraries exposed to both 3× and 10× the EC_50_ value of GSK3186899A. Similarly, the selection of the expanded library with DNDI-6174 (3× EC_50_) enriched DDD00107332-resistant promastigotes, with these parasites demonstrating considerable fitness under selection at this level (90%). Once again, these cross-resistant phenotypes were reproducible in follow-up EC_50_ determinations with these cell lines in individual culture (Table S5). Since the physiological function of CRK12 in *Leishmania* has yet to be determined, speculating on the basis for this apparent reciprocal cross-resistance relationship is difficult. Understand the mechanisms underpinning this association will be the focus of our future studies. Nevertheless, these data suggest that CRK12, and cytochrome *b* inhibitors may not be suitable partners in any future VL combination therapy.

### Conclusions

Future elimination of VL will require the development of novel anti-leishmanials with diverse mechanisms of action. Multiple and diverse treatment options will give the best possible chance of developing combination therapies that can overcome the threat posed by emerging drug resistance. To ensure diversity of treatment options in the future, it is paramount that the mechanisms of action of compounds at various stages of the development pipeline are understood and compounds acting against the same molecular targets rationalized. Development of the screening platform described in this study, which we intend to call RES-Seq, will accelerate the discovery of anti-leishmanial compounds that act via previously established mechanisms of action or are vulnerable to known mechanisms of resistance. The library is eminently scalable, and we will continue to add compound-resistant cell lines generated via *in vitro* evolution or gene editing. One significant advantage is that once composite libraries have been assembled, multiple stabilates can be prepared and used to expedite future screens. This barcoded library is now routinely used in our lab to profile new compounds in development.

Undoubtedly, RES-Seq does have limitations. In the current format, we are limited to screening compounds that have demonstrated activity against promastigotes and would not be suitable for screening of compounds only active against amastigotes. In our experience, there are relatively few amastigote-specific compounds in current drug discovery pipelines; nevertheless, we are in the process of adapting the screen to address this issue. Specifically, investigating the possibility of screening test compounds against host macrophages infected with barcoded library cell lines. A long-term goal will be to assess our barcoded library of parasites in mouse models of infection to assess the fitness cost of specific mutations in a disease-relevant context. Adding barcoded drug-resistant clinical isolates to the library was considered but rejected since we rationalized that their inherent genetic diversity would make interpreting relative fitness extremely challenging. A better strategy may be to genetically engineer mutations confirmed as playing a role in clinical resistance into our current wild-type, parental cell line.

Going forward, we believe that RES-Seq will become an important tool to ensure that anti-leishmanial compounds with diverse mechanisms of action are prioritized for development. We hope these studies will also act as a template for researchers looking to monitor the growth kinetics of pooled parasite cell lines under pressure from a common stressor or selective agent.

## MATERIALS AND METHODS

### Compounds

All the compounds used in this study were kindly provided by GSK, DND*i* or the Drug Discovery Unit, University of Dundee. The purity of each compound was confirmed by LC-MS to be >95%.

### Drug sensitivity assays

Drug sensitivity assays were carried out as previously described (16). Data were processed using GRAFIT (version 5.0.4, Erithacus Software) and fitted to a two-parameter equation to determine the effective concentration inhibiting growth by 50% (EC_50_):


$$y=\frac{100}{1+{\left(\frac{\left[I\right]}{EC_{50}}\right)}^{m}}$$

In this equation, [*I*] represents the inhibitor concentration, and *m* is the slope factor. Experiments were performed at least in three independent biological replicates with the data presented as the weighted mean ± standard deviation.

### Cell lines and culture conditions

All cell lines investigated in this study were generated from a clonal *Leishmania donovani* cell line *Ld*BOB (derived from MHOM/SD/62/1S-CL2D). Throughout, parasites were grown as promastigotes at 28°C, as previously described (18).

### Generation of resistant clones

All compound-resistant parasites reported in this study were generated in an identical manner, namely by subculturing clones of *Ld*BOB in the continuous presence of test compounds. Starting at sublethal concentrations, drug concentrations in at least three independent cultures were increased in a stepwise manner, usually by twofold. When parasites were able to survive and grow in concentrations of drug equivalent to 20× the established EC_50_ value, the resulting cell lines were cloned by limiting dilution in the presence of compound. Drug sensitivity assays following culture of these clones in the absence of drug selection for 20 passages confirmed that in all cases resistance was stable. It should be noted that resistant clones were grown in the absence of drug selection prior to combined library assembly. Genomic DNA was isolated from all resistant clones, then whole genome sequenced using a HiSeq4000 or DNBseq next-generation sequencing platform (Beijing Genomics Institute, Hong Kong), as previously described (12). Specific details of the resulting resistance-conferring genomic changes identified via this analysis have been previously published for the vast majority of resistant clones reported in this study (summarized in Table 1). Four resistant clones that have not been previously reported (Amph B-Res 1 and 3, DNDI-6690-Res 2, and BPQ-Res 3) were included in our barcoded library. Full details of these clones, selected with amphotericin B, DNDI-6690, and buparvaquone, respectively, will be focus of subsequent publications. However, the specific mutations confirmed to drive resistance in these clones are reported (Table 1).

### Generation of transgenic cell lines

All transgenic cell lines (knockout, overexpression, and gene-edited) generated to confirm the role of the genomic changes identified in our resistant clones have been reported previously with the exception of DNM1^G606V^, which was generated in a WT background constitutively expressing Cas9 and T7 RNA polymerase as previously described (17, 25). Briefly, the sgRNA template that directed Cas9 to cleave *DNM1* (LdBPK_292310.1) at nucleotide 1813 was generated by PCR amplification of oligo LBT-021 (5′-gaaattaatacgactcactatagggGCCATTCGCGAGATGGTGGAgttttagagctagaaatagc-3′) with primer G00 (5′-AAAAGCACCGACTCGGTGCCACTTTTTCAAGTTGA TAACGGACTAGCCTTATTTTAACTTGCTATTTCTAGCTCTAAAAC-3′), using the protocol established by Gluenz and colleagues (26) and was transfected into *L. donovani* promastigotes along with 1 nmol repair oligo LBT-020 (5′-AGTACATGAACAGCGCCATTCGCGAAATGGTCGAGGTCTATTTTTCGATTGTGAAGGGCAAC-3′) containing the desired mutations (G1817T, which confers the G606V amino acid change, as well as synonymous mutations G1806A and G1812C) as described previously (17). DNDI-6690 was added at 100 nM 24 h later. Drug-selected cells were cloned by limiting dilution. The presence of the G1817T mutation conferring G606V along with synonymous mutations G1806A and G1812C was confirmed by PCR amplification of *DNM1* from clones using oligos LBT-028 (5′-TTAATACCCGGGATGGACCAGTTGATCAGCG-3′) and LBT-029 (5′-TTAATATCTAGATTAGGCGCCGGCTTGCATGGATGAGG-3′) and subsequent Sanger sequencing with LBT-029.

### Barcode design and synthesis

Barcodes were designed comprising two 7-nucleotide variable sequences (variables 1 and 2), separated, and flanked by “TAG” sequences. To facilitate subsequent cloning, barcodes maintained XmaI and XbaI cleavage sites at their 5´ and 3´ends, respectively (Fig. S1). All possible 7-nucleotide permutations (4^7^) were generated using a quaternary (base 4) counting formula where 0, 1, 2, and 3 were substituted for A, C, G, and T, respectively. Undesirable barcodes, such as those maintaining >2 identical nucleotides in a row or encoding undesirable restriction sites, were eliminated. The remaining 6,350 barcodes were randomized in preparation for synthesis.

To generate forward and reverse barcode pairs (50 µM, Thermo), barcodes were combined in hybridization buffer (100 mM potassium acetate/30 mM HEPES pH 7.4) and annealed by heating to 95°C for 5 min followed by slow cooling to RT. Annealed barcodes were then combined, diluted to 6 ng/µL, and ligated into the *Leishmania*-specific vector pIR1-SAT pre-digested with XmaI and XbaI. Following transformation into *E. coli* DH5α, single colonies were selected, and barcode-containing plasmids recovered. Barcode sequences were read by Sanger sequencing using LBT-001 (5′-TTTCAAGGCTTCCCGAACG-3′) as a sequencing primer.

### Barcoding of cell lines

To barcode WT, drug-resistant, and transgenic cell lines, promastigotes (10^7^) from each cell line were transfected with barcoded plasmids (5–10 µg, one barcode per cell line), using reagents from the Human T-cell Nucleofector Kit (Lonza) and the Amaxa Nucleofector (program V-033), as described previously (21). Promastigotes bearing barcodes were selected by continuous culture in the presence of nourseothricin (100 µg/mL).

### Proof-of-concept screen

Twelve barcoded cell lines (WT plus 11 drug-resistant cell lines) were combined to a final density of 10^5^ cells/mL and dispensed into T25 flasks (10 mL). This combined library was then selected with a number of compounds with known mechanisms of action (summarized in Table 1) at concentrations equivalent to 3× and 10× their established EC_50_ values; a no-drug control culture was maintained alongside. Cell densities were monitored daily, and once they exceeded 8 × 10^6^ parasites/mL, cells were sub-cultured back to 10^5^ cells/mL in fresh media with fresh compound selection. The residual cells following sub-culture were harvested by centrifugation (1,912 × *g*, RT, 10 min), and DNA extracted using a standard protocol. The library was grown under compound selection for three sub-cultures or a maximum of 16 days.

### Barcode analysis—proof-of-concept screen

Barcodes were PCR amplified from 100 ng extracted DNA using Q5 polymerase (NEB) and primers LBT-056 (5′-CGCGTGCACATCATCAACTGTCTCTTGTCGG) and LBT-057 (5′-GATTCACAGCGCGCCTGCTCGTCC). PCR products were Sanger sequenced (University of Dundee DNA Sequencing Facility) using primer LBT-001, and barcodes were identified from sequencing chromatograms.

### Barcoded library—expanded library screen

The complexity of our initial validation library was expanded further through the addition of six barcoded resistant and transgenic cell lines (summarized in Table 1). All cell lines were combined into a single composite library, as described for the proof-of-concept library. For the clinical drug candidate screen described below, the expanded library was screened immediately. At this point, multiple aliquots of the library were also prepared and stored at −80°C for use in future screens.

Anti-leishmanial clinical drug candidates were screened against the expanded library essentially as described above. However, in this instance, parasites were harvested, and DNA prepared after a single sub-culture (typically on days 3–6 of drug exposure). At this point, cultures were also sub-cultured, and growth under compound selection monitored for a further 3 days to assess the growth rate recovery.

### Analysis of recovered barcodes via Illumina sequencing

Barcodes were PCR-amplified from harvested DNA (200 ng) using GoTaq G2 master mix (NEB, 100 µL reactions) and primers LBT-056 and LBT-057. Typically, a total of 5–10 µg amplified DNA was recovered from these reactions. Amplified DNA (5–10 μg in 100 µL Tris-buffer) was sequenced using an Illumina HiSeq or DNBseq platform (Beijing Genomics Institute). The abundance of each barcode in each sample was determined from reads using a custom script developed in Python, within a Jupyter notebook environment (https://jupyter.org). The script operates by identifying the barcode sequences within the paired reads, considering both forward and reverse complement directions. The Python script has been made publicly accessible in GitHub (https://github.com/mtinti/RES-Seq) and has also been preserved in Zenodo (10.5281/zenodo.8145218).

### Data analysis

The number of barcode reads for each drug condition was subsequently analyzed in Excel (Extended data sets I and II). The library composition after each sub-culture was determined as the proportion of each barcode, expressed as the percentage of the total barcode reads. This information was used to determine the doubling time of each cell line in the library. The fitness of each cell line was calculated as 1/doubling time relative to uninhibited WT (for cell line fitness) or to the corresponding uninhibited resistant line (for fitness retention in the presence of drug).

## Data Availability

Whole-genome sequencing data have been deposited with the National Center for Biotechnology Information Sequence Read Archive (NCBI SRA) under the following project codes: PRJNA994717 (BPQ-Res 3), PRJNA994718 (DNDI-0690-Res 2), and PRJNA994719 (Amph B-Res 1 and Amph B-Res 3).

## SUPPLEMENTAL MATERIAL

Supplemental figures and tables mbio.01803-23-s0001.docx Fig. S1 to S5 and Tables S1 to S6.

Extended dataset I mbio.01803-23-s0002.xlsx Composite data sets and data processing for proof-of-concept library screens.

Extended dataset II mbio.01803-23-s0003.xlsx Composite data sets and data processing for expanded library screens.

ASM does not own the copyrights to Supplemental Material that may be linked to, or accessed through, an article. The authors have granted ASM a non-exclusive, world-wide license to publish the Supplemental Material files. Please contact the corresponding author directly for reuse.
