# Supplementary material for: RES-Seq—a barcoded library of drug-resistant Leishmania donovani allowing rapid assessment of cross-resistance and relative fitness
Source: mBio. 2023 Nov 6;14(6):e01803-23. doi: 10.1128/mbio.01803-23 (PMC10746238; doi:10.1128/mbio.01803-23)
Supplement: Supplemental figures and tables — Fig. S1 to S5 and Tables S1 to S6. [file mbio.01803-23-s0001.docx]

**SUPPLEMENTARY INFORMATION**

**RES-Seq – a barcoded library of drug-resistant *Leishmania* *donovani* allowing rapid assessment of cross-resistance and relative fitness**

Lindsay B. Tulloch^1^, Sandra Carvalho^1^, Marta Lima^1^, Richard J. Wall^1*^, Michele Tinti^1^, Erika Pinto^2^, Lorna MacLean^2^ and Susan Wyllie^1#^

^1^Wellcome Centre for Anti-Infectives Research, School of Life Sciences, University of Dundee, Dow Street, Dundee DD1 5EH, United Kingdom.

^2^Drug Discovery Unit, Wellcome Centre for Anti-infectives Research, University of Dundee, Dow Street, Dundee, DD1 5EH, UK

^*^Current address: London School of Hygiene and Tropical Medicine, Keppel St, London WC1E 7HT, UK

^#^**Corresponding author:** Susan Wyllie ([s.wyllie@dundee.ac.uk](mailto:s.wyllie@dundee.ac.uk))

**Running title:** Barcoding of drug-resistant *Leishmania* *donovani*

**Supplementary information**

**
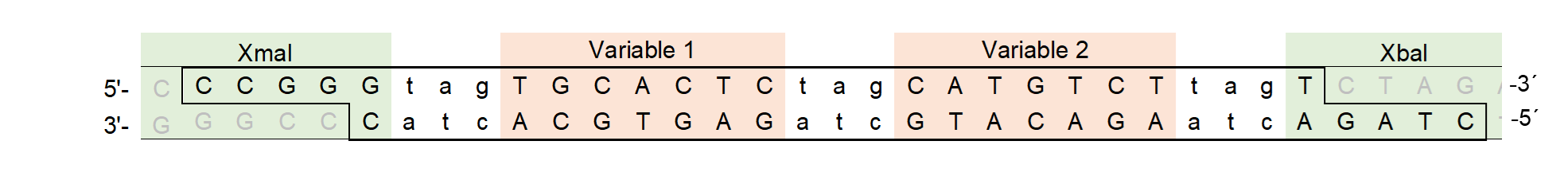
**

**Fig. S1: Barcoding of resistant cell lines.** Graphical representation of the barcodes inserted into the XmaI/XbaI restriction sites of the pIR1-SAT plasmid. Barcodes consist of two 7-bp variable sequences flanked by ‘TAG’ motifs to facilitate barcode alignment.

**
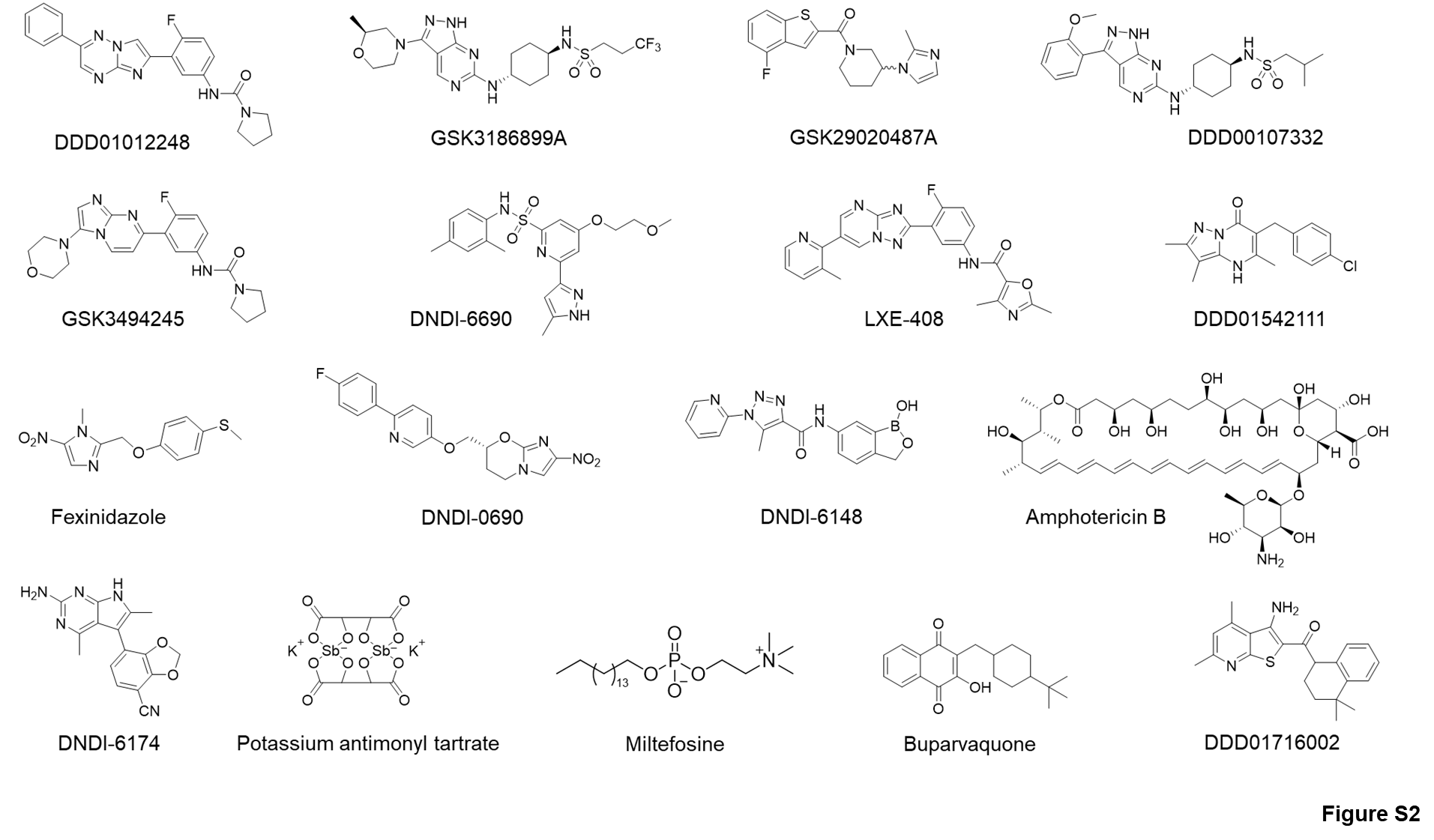
**

**Fig. S2: Chemical structures of compounds and drugs used in this study.**

**
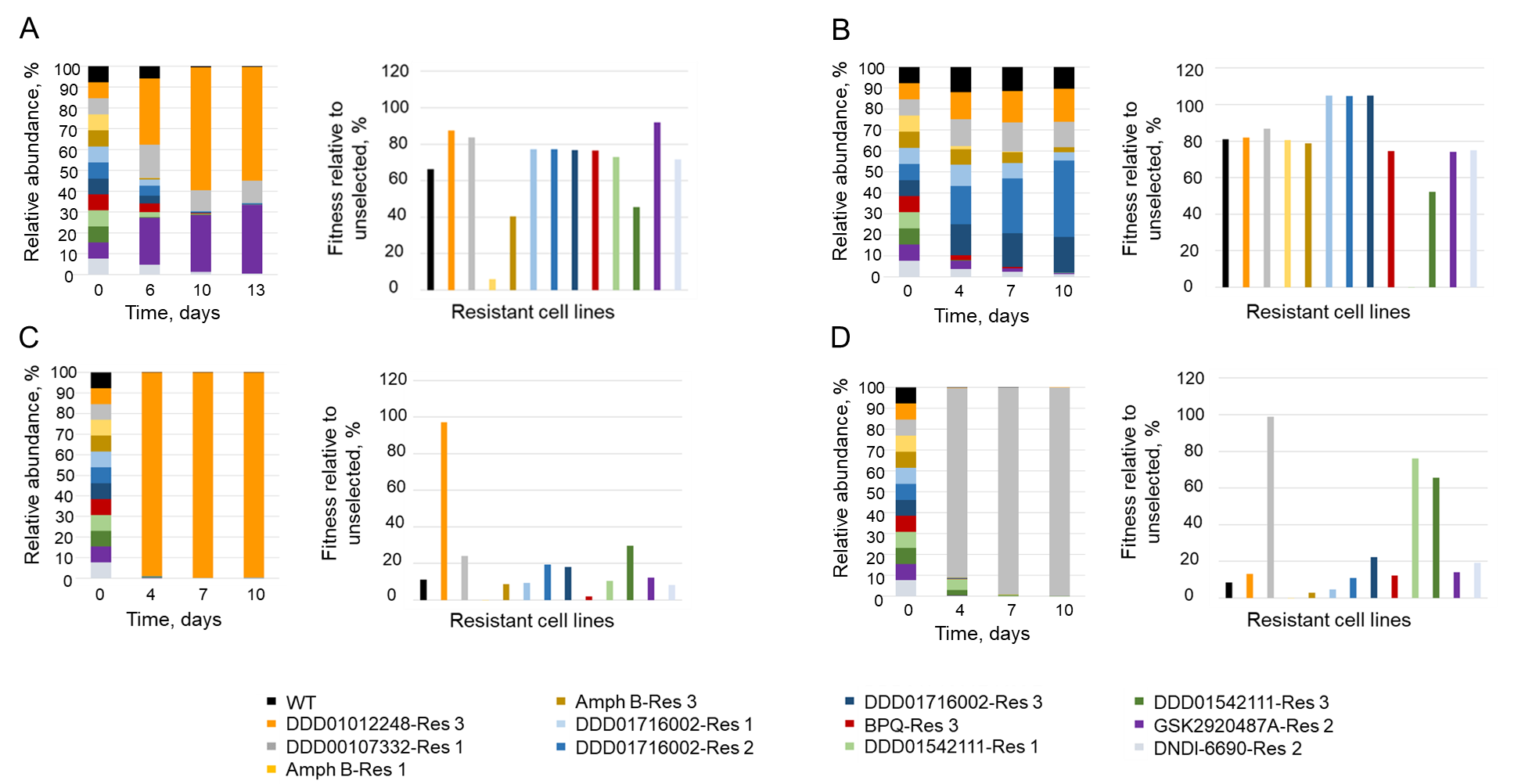
**

**Fig. S3: Compound selection of the proof-of-concept barcoded library.** Library composition (%) over time and fitness profiling of cell lines (relative to unselected) following selection (1 passage) with GSK2920487A (A), DDD01716002 (B), DDD01012248 (C), and DDD00107332 (D) at 3× their respective EC_50_ values. Colour coded key to identify each cell line within the library provided.


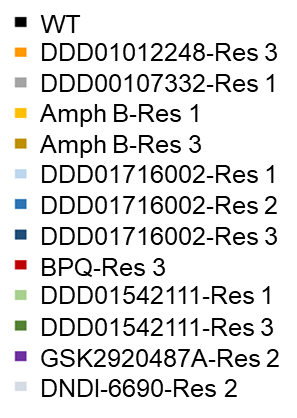
**
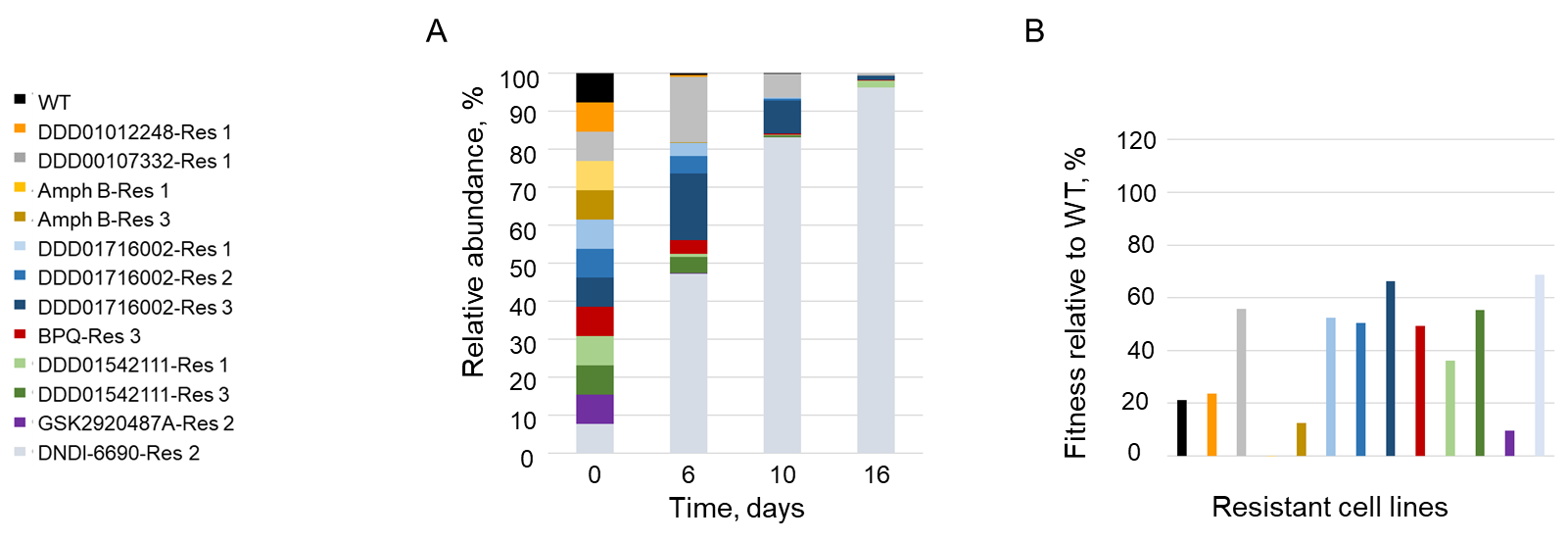
**

**Fig. S4: Compound selection of the proof-of-concept barcoded library with *N*-myristoyltransferase inhibitor DDD85646.** Library composition (%) over time (A) and fitness profiling of cell lines (relative to unselected) following selection (1 passage) (B) with DDD85646 at 3× its EC_50_ value. Colour coded key to identify each cell line within the library provided.


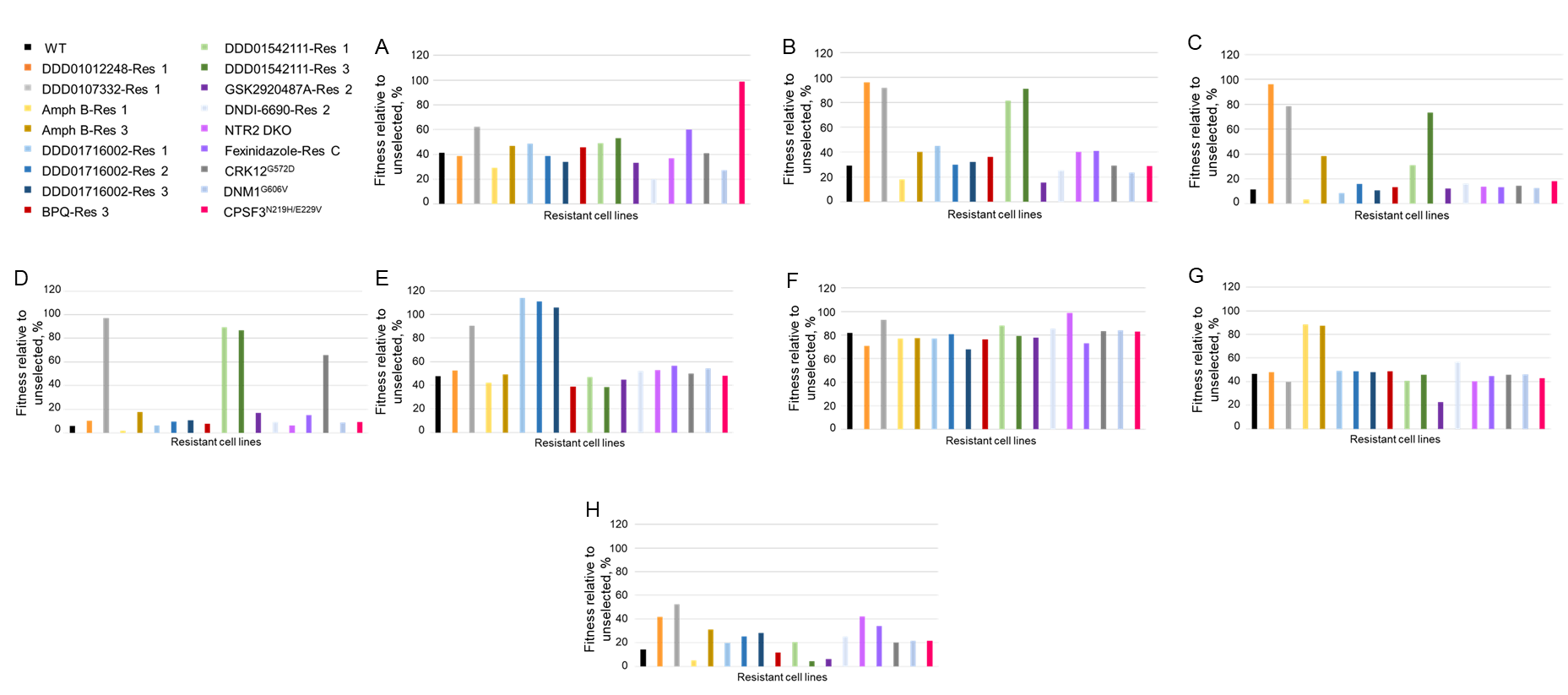

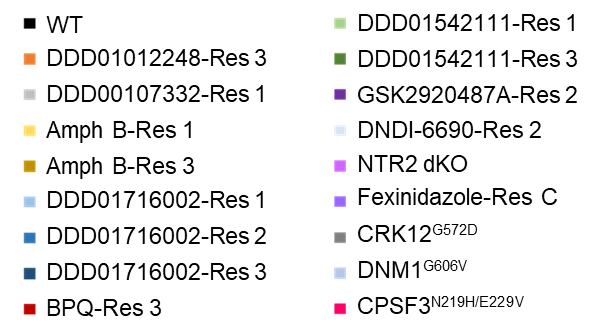


**Fig. S5: Selection of the expanded barcoded library with VL clinical and preclinical drug candidates.** Fitness profiling of cell lines within the library relative to unselected cell lines following selection (1 passage) with DNDI-6148 (A), GSK3494245 (B), LXE-408 (C), GSK3186899A (D), DNDI-6174 (E), DNDI-0690 (F), amphotericin B (G) and potassium antimonyl tartrate (H) at 3× their respective EC_50_ values. Colour coded key to identify each cell line within the library provided.

**Table S1: Unique identifier barcodes assigned to resistant cell lines included in the proof-of-concept and expanded pooled libraries.**

| **Cell line** | **Barcode variable sequences** | |
| --- | --- | --- |
|  | **Position 1** | **Position 2** |
| WT | CATCAGA | TGCTGAG |
| DDD01012248-Res 1 | TATGGCG | CGCAATA |
| DDD00107332-Res 1 | GCTTCCA | ATCCTTG |
| CRK12^G572D^ | CCTACAC | TTCGTGT |
| Amph B-Res 1 | AGTGGCT | GACAATC |
| Amph B-Res 3 | TTCGCGT | CCTATAC |
| GSK2920487A-Res 2 | ATGGAAT | GCAAGGC |
| DNDI-6690-Res 2 | GCGTGGA | ATACAAG |
| DNM1^G606V^ | GCAAGAG | ATGGAGA |
| CPSF3^N219H/E229V^ | AGACAAC | GAGTGGT |
| DDD01716002-Res 1 | GATCGAC | AGCTAGT |
| DDD01716002-Res 2 | CGCAATC | TATGGCT |
| DDD01716002-Res 3 | CAACCAT | TGGTTGC |
| BPQ-Res 3 | TCGCTCG | CTATCTA |
| DDD01542111-Res 1 | TGCACTC | CATGTCT |
| DDD01542111-Res 3 | CTCCTAC | TCTTCGT |
| NTR2 DKO | CACAGCT | TGTGATC |
| Fexinidazole-Res C | ATAACTG | GCGGTCA |

**Table S2: Potency of study compounds against *L. donovani* WT promastigotes.**EC_50_ values are the weighted mean ± standard deviation of at three biological replicates (n = 3), with each biological replicate comprised of two technical replicates.

| **Compound** | **EC_50_, nM (WT)** |
| --- | --- |
| DDD01012248 | 6.3 ± 0.4 |
| GSK3494245 | 120 ± 3 |
| LXE-408 | 7.9 ± 1 |
| GSK3186899A | 25 ± 2 |
| DDD00107332 | 0.8 ± 0.03 |
| Amphotericin B | 19 ± 0.1 |
| GSK2920487A | 500 ± 20 |
| DNDI-6690 | 23 ± 2 |
| DNDI-6174 | 18 ± 0.6 |
| DDD01716002 | 24 ± 2 |
| Buparvaquone | 33 ± 2 |
| DDD01542111 | 30 ± 1 |
| DNDI-6148 | 680 ± 28 |
| DNDI-0690 | 11 ± 1 |
| Fexinidazole | 13,000 ± 600 |
| Potassium antimonyl tartrate | 30,000 ± 670 |
| Miltefosine | 13,000 ± 1900 |

| **Cell line** | **Compound** | **Fold resistance relative to WT** |
| --- | --- | --- |
| DDD01012248-Res 1 | DDD01012248 | 20 |
| DDD00107332-Res 1 | DDD00107332 | 82 |
| CRK12^G572D^ | DDD00107332 | 3 |
| Amph B-Res 1 | Amphotericin B | 63 |
| Amph B-Res 3 | Amphotericin B | 8 |
| GSK2920487A-Res 2 | GSK2920487A | 11 |
| DNDI-6690-Res 2 | DNDI-6690 | 29 |
| DNM1^G606V^ | DNDI-6690 | 9 |
| CPSF3^N219H^ | DNDI-6148 | 3 |
| DDD01716002-Res 1 | DDD01716002 | 11 |
| DDD01716002-Res 2 | DDD01716002 | 7 |
| DDD01716002-Res 3 | DDD01716002 | 13 |
| BPQ-Res 3 | Buparvaquone | 218 |
| DDD01542111-Res 1 | DDD01542111 | 51 |
| DDD01542111-Res 3 | DDD01542111 | 48 |
| NTR2 DKO | Delamanid | >250 |
| Fexinidazole-Res C | Fexinidazole | >70 |

**Table S3: Resistant cell lines included in the proof-of-concept and expanded pooled libraries.** Compounds used in the evolution of resistance are noted. The fold-resistance these cell lines demonstrated relative to WT are indicated. EC_50_ values are the weighted mean ± standard deviation of three biological replicates (n = 3), with each biological replicate comprised of two technical replicates.

**Table S4: Comparative doubling times of cell lines in the barcoded library and individual culture**. Doubling times calculated from RES-Seq data (also see **Extended dataset I**) and from individual cell lines in culture.

| **Cell line** | **Doubling time, h** | |
| --- | --- | --- |
|  | **Barcoded library** | **Individual culture** |
| WT | 8.7 | 8.2 |
| DDD01012248-Res 3 | 8.7 | 8.2 |
| DDD00107332-Res 1 | 8.9 | 8.5 |
| Amph B-Res 1 | 12.7 | 13.9 |
| Amph B-Res 3 | 9.4 | 8.7 |
| DDD01716002-Res 1 | 11.8 | 10.9 |
| DDD01716002-Res 2 | 10 | 8.6 |
| DDD01716002-Res 3 | 10.2 | 9.2 |
| BPQ-Res 3 | 10.8 | 9.9 |
| DDD01542111-Res 1 | 10.8 | 10.3 |
| DDD01542111-Res 3 | 11.1 | 11.2 |
| GSK2920487A-Res 2 | 10 | 9.1 |
| DNDI-6690-Res 2 | 9.8 | 8.7 |

**Table S5: Comparison of calculated cell line fitness relative to WT across multiple libraries.** Data is collated from libraries grown in the absence of drug selection.

| Cell line | Calculated fitness retention | | | | |  |
| --- | --- | --- | --- | --- | --- | --- |
|  | Rep 1 | Rep 2 | Rep 3 | Rep 4 | **AV** | SD |
| WT | 100 | 100 | 100 | 100 | **100** | - |
| DDD01012248-Res 1 | 100 | 100 | 101 | 101 | **101** | 1 |
| DDD00107332-Res 1 | 94 | 101 | 99 | 95 | **97** | 3 |
| Amph B-Res 1 | 68 | 70 | 68 | 74 | **70** | 3 |
| Amph B-Res 3 | 95 | 93 | 90 | 87 | **91** | 3 |
| DDD01716002-Res 1 | 75 | 76 | 71 | 74 | **74** | 2 |
| DDD01716002-Res 2 | 82 | 88 | 90 | 92 | **88** | 4 |
| DDD01716002-Res 3 | 80 | 88 | 89 | 90 | **87** | 5 |
| BPQ-Res 3 | 80 | 79 | 83 | 86 | **82** | 3 |
| DDD01542111-Res 1 | 76 | 82 | 83 | 88 | **82** | 5 |
| DDD01542111-Res 3 | 74 | 79 | 81 | 86 | **80** | 5 |
| GSK2920487A-Res 2 | 90 | 83 | 87 | 86 | **87** | 3 |
| DNDI-6690-Res 2 | 89 | 87 | 92 | 89 | **89** | 2 |
| NTR2 DKO | - | 92 | 94 | 93 | **93** | 1 |
| FxR Res 1 | - | 75 | 81 | 81 | **78** | 4 |
| CRK12^G572D^ | - | 100 | 100 | 98 | **100** | 2 |
| DNM1^G606V^ | - | 103 | 101 | 101 | **102** | 1 |
| CPSF3^N219H/E229V^ | - | 98 | 101 | 100 | **100** | 1 |

**Table S6: EC_50_ fold-changes relative to WT.** Confirmatory EC_50_ values were determined for each cell line in individual culture. Data represents mean values from biological replicates (n ≥ 3).

| **Cell line** | **DDD00107332** | **DDD01542111** | **GSK3186899A** | **DNDI-6174** |
| --- | --- | --- | --- | --- |
| DDD00107332-Res 1 | 85 | 15 | 34 | 3 |
| DDD01542111-Res 1 | 5 | 50 | 5 | 0.4 |
| DDD01542111-Res 3 | 5 | 40 | 3 | 0.6 |

**Extended dataset I: Composite datasets and data processing for proof-of-concept library screens.** See attached (XLSX).

**Extended dataset II: Composite datasets and data processing for expanded library screens.** See attached (XLSX).
